# Maternal Dietary Patterns during Pregnancy and Their Association with Gestational Weight Gain and Nutrient Adequacy

**DOI:** 10.3390/ijerph17217908

**Published:** 2020-10-28

**Authors:** Naomi Cano-Ibáñez, Juan Miguel Martínez-Galiano, Miguel Angel Luque-Fernández, Sandra Martín-Peláez, Aurora Bueno-Cavanillas, Miguel Delgado-Rodríguez

**Affiliations:** 1 Department of Preventive Medicine and Public Health, University of Granada, 18071 Granada, Spain; ncaiba@ugr.es (N.C.-I.); sandramartin@ugr.es (S.M.-P.); abueno@ugr.es (A.B.-C.); 2 Consortium for Biomedical Research in Epidemiology and Public Health (CIBERESP), 28029 Madrid, Spain; miguel-angel.luque@lshtm.ac.uk (M.A.L.-F.); mdelgado@ujaen.es (M.D.-R.); 3 Instituto de Investigación Biosanitaria (ibs. GRANADA), Complejo Hospitales Universitarios de Granada/Universidad de Granada, 18071 Granada, Spain; 4 Department of Nursing, University of Jaén, 23071 Jaén, Spain; 5 Department of Noncommunicable Disease Epidemiology, London School of Hygiene and Tropical Medicine, London WC1H 9SH, UK; 6 Division of Preventive Medicine and Public Health, University of Jaén, 23071 Jaén, Spain

**Keywords:** maternal dietary patterns, pregnancy, gestational gain weight, offspring

## Abstract

Several epidemiologic studies have shown an association between Gestational Weight Gain (GWG) and offspring complications. The GWG is directly linked to maternal dietary intake and women’s nutritional status during pregnancy. The aim of this study was (1) to assess, in a sample of Spanish pregnant women, the association between maternal dietary patterns and GWG and (2) to assess maternal dietary patterns and nutrient adequate intake according to GWG. A retrospective study was conducted in a sample of 503 adult pregnant women in five hospitals in Eastern Andalusia (Spain). Data on demographic characteristics, anthropometric values, and dietary intake were collected from clinical records by trained midwives. Usual food intake was gathered through a validated Food Frequency Questionnaire (FFQ), and dietary patterns were obtained by principal component analysis. Nutrient adequacy was defined according to European dietary intake recommendations for pregnant women. Regression models adjusted by confounding factors were constructed to study the association between maternal dietary pattern and GWG, and maternal dietary patterns and nutritional adequacy. A negative association was found between GWG and the Mediterranean dietary pattern (crude β = −0.06, 95% CI: −0.11, −0.04). Independent of maternal dietary pattern, nutrient adequacy of dietary fiber, vitamin B9, D, E, and iodine was related to a Mediterranean dietary pattern (*p* < 0.05). A Mediterranean dietary pattern is related to lower GWG and better nutrient adequacy. The promotion of healthy dietary behavior consistent with the general advice promoted by the Mediterranean Diet (based on legumes, vegetables, nuts, olive oil, and whole cereals) will offer healthful, sustainable, and practical strategies to control GWG and ensure adequate nutrient intake during pregnancy.

## 1. Introduction

Excess body weight during pregnancy is a public health concern owing to its high prevalence; increased risk of maternal diseases, such as gestational diabetes; and delivery complications [1]. Even though the current maternal guidelines stress the need for Gestational Weight Gain (GWG) control [2], the 2011 US pregnancy nutrition surveillance system has shown that up to 47% of pregnant women had excessive GWG and 23% had inadequate GWG according to the recommendations provided by the Institute of Medicine (IOM) [3]. 

GWG, or the total amount of weight gained in pregnancy, is a complex physiologic response to accommodate the natural responses to pregnancy, such as gestational fat deposition and fetal growth [4]. Several epidemiological studies have shown an association between GWG and offspring complications. Women who gain excessive weight during pregnancy (i.e., more than the amount recommended in guidelines) are more likely to have infants with high birth weight, premature delivery, and infants with an increased risk of developing childhood obesity [5]. Furthermore, excessive GWG is associated with an increased risk of maternal diseases, such as gestational diabetes mellitus and preeclampsia [6]. On the other hand, women with insufficient GWG are more likely to have infants with low birth weight and intrauterine growth retardation [7].

Nevertheless, GWG is a potentially modifiable risk factor because it is directly linked to maternal nutritional habits during pregnancy. Current research evaluating GWG according to dietary intake has focused on isolated foods [8,9] or nutrients [10,11] instead of the dietary pattern of food consumption. The analysis of dietary patterns could provide a better understanding of maternal dietary food intake and thus of women’s nutritional status during pregnancy. Although a correct diet is essential to maintain an adequate nutritional status at all stages of life, during pregnancy, nutritional needs are increased in order to meet fetal requirements, especially for some micronutrients. In particular, essential micronutrients are necessary to prevent maternal and perinatal adverse health conditions. However, a deficient intake of essential micronutrients is commonly reported [12] and is associated with an increased nutritional vulnerability in pregnant women [13,14], specifically among those women with worse dietary food intake. Usually, this deficient intake is linked to occidental dietary patterns characterized by a high intake of meat or meat products; snacks; baked desserts; and sugar-sweetened beverages, providing large amounts of saturated fatty acids and simple carbohydrates as added sugars [15].

There is a scarcity of findings regarding dietary factors, nutritional adequacy, and GWG. Furthermore, inadequate GWG and nutrient intake during pregnancy has a negative impact on maternal, perinatal, and fetal health [16]. Therefore, we aimed (1) to assess the association of maternal dietary patterns with GWG and (2) to analyze the association between maternal dietary patterns and nutrient adequate intake according to GWG among Spanish pregnant women.

## 2. Methods

### 2.1. Study Design, Settings, and Participants

**Figure 1:**
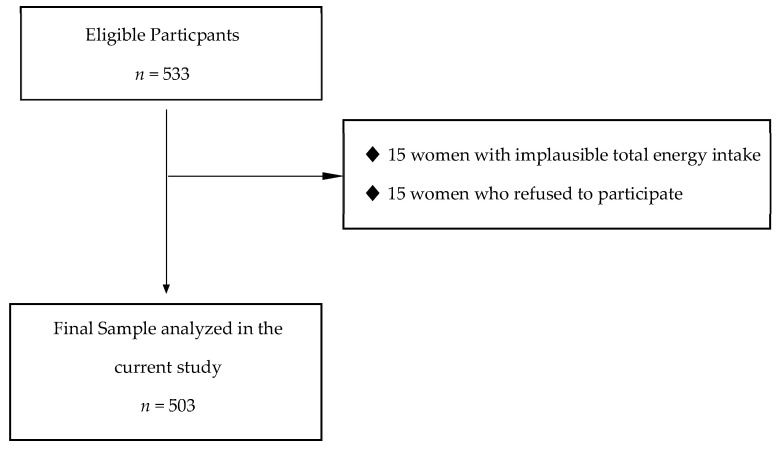
Study Flow-Chart.

We designed a retrospective, observational study. Data were obtained from a cohort of pregnant women attending five hospitals in Eastern Andalusia (Spain). Women were recruited from 15 May 2012 through 15 July 2015. Eligible participants were women who resided in the referral area of the five hospitals located in the provinces of Jaen (2 hospitals), Granada (2 hospitals), and Almeria (1 hospital), who understood the Spanish language, gave birth to a single live newborn, and agreed to complete and return the Food Frequency Questionnaire (FFQ) after delivery assessing their dietary intake during pregnancy. After applying the inclusion criteria, 533 women were included in the study. Fifteen women refused participation, and 15 women were excluded because they presented energy intakes outside of predefined limits [17]. A sample of 503 women was analyzed in the current analysis (Figure 1).

The ethics committees from the hospitals approved the study protocol. All women included in this study filled out informed consent and data treatment forms to enroll in the study, following the ethical standards of institutions where they were identified.

### 2.2. Data Collection and Outcomes

Data were collected retrospectively on anthropometric measures and dietary food and energy intake. Deficient dietary patterns of food consumption and excessive GWG were identified based on IOM guidelines. Next, we evaluated the quality of diet, based on nutrient adequate intake according to recommendations for pregnant women. The primary outcome of the study was maternal GWG and adequacy of nutrient intake, with maternal dietary patterns as the main exposure. 

#### 2.2.1. Dietary Assessment

Trained midwives collected dietary intake information using a 137 item semiquantitative FFQ, which was given to women after delivery. This FFQ has been previously translated, adapted, and validated in Spanish women 18 to 74 years of age [18,19]. The FFQ provided a list of foods commonly used by the Spanish population and inquired about the consumption of these foods during the previous year. For each food item in the FFQ, women were asked to report the frequency of consumption and portion size. The FFQ included nine response options (never or almost never, 1–3 times a month, once a week, 2–4 times a week, 5–6 times a week, once a day, 2–3 times a weekday, 4–6 times a day, and more than six times a day). The dietary intake in grams per day was estimated using the indicated frequencies of consumption that were converted to intakes per day and multiplied by the weight of the standard serving size. Nutrient information, as well as total energy intake, was derived from Spanish food composition tables [20,21]. After computing total energy intake, 15 women were excluded because of implausible extreme energy intakes (<500 kcal/day and >3500 kcal/day) [17], leaving 503 women for analysis. Finally, food intake was adjusted for total energy intake using the residual method proposed by Willet et al. [22]. 

#### 2.2.2. Dietary Pattern Construction

Factor analysis has been extensively used to detect common patterns among highly correlated variables through the use of FFQ [23]. This methodology is a tool commonly employed to extract posteriori dietary patterns [24]. We applied factor analysis with the Principal Components Method (PCA). First, the food items of the FFQ were combined into 16 groups by similar nutrient profile and culinary usage. A detailed description of each food group is reported in Table 1. The daily intake (in grams) for each food group, adjusted by total energy intake, was used in the factor analysis to identify maternal dietary patterns. On the basis of the values of the factor loadings, two main dietary patterns were defined, characterized by high factor loadings of specific food groups.

#### 2.2.3. Anthropometry

Pre-pregnancy weight was self-reported by pregnant women during the first appointment with the midwife. During each antenatal appointment, maternal weight (in kg) and height (in cm) were measured at the antenatal outpatient clinic, and maternal weight (in kg) before delivery was measured at delivery admission by a midwife. Total GWG (in kg) was obtained as the difference in maternal weight between pre-pregnancy weight and weight at delivery admission, as other authors have done previously [25]. Finally, according to the IOM guidelines, we defined the GWG as 12.5–18 kg for underweight, 11.5–16 kg for normal or adequate weight, 7–11.5 kg for overweight, and 5–9 kg for obese women. Weight gain below or above the recommended range was considered as inadequate or excessive GWG. 

#### 2.2.4. Diet Quality: Nutrient Adequate Intake

The dietary intake of a selection of nutrients, including dietary fiber, vitamins A, B_9_, B_12_, D, and E, and minerals such as calcium, phosphorus, magnesium, iron, iodine, potassium, selenium, and zinc, was compared with the recommended intakes for these nutrients in pregnant women according to the criteria established by the European Food Safety Agency (EFSA) [26]. Considering the Average Requirements (AR) and/or Average Intake (AI) and Upper-Level intake (UL), we built three categories: (i) deficit intake (intake below AR/AI), (ii) adequate intake (intake between AR/AI and UL), and (iii) excessive intake (intake higher UL). To decrease potential measurement errors derived from the use of the FFQ (overestimation bias), we calculated the proportion of women with intakes below two thirds (2/3) of the Dietary Reference Intakes (DRIs), as other authors have reported previously [14,27]. Results were based on dietary intake data only, excluding supplements. 

#### 2.2.5. Other Maternal Variables Related to Patient Characteristics

The assessment of data was obtained via three different sources: (i) personal interviews carried out by trained midwives in the hospital within the two days after giving birth, (ii) clinical records, and (iii) prenatal care records. Information was obtained on general sociodemographic characteristics, including maternal age at pregnancy, lifestyle habits, and personal characteristics. Social class ranged from I (the highest) to V (the lowest), coded according to the classification of the Spanish Society of Epidemiology [28], which is similar to that reported in the Black Report [29]. We categorized smoking status during pregnancy as a current smoker and non-smoker (including previous smokers). Finally, we also computed the number of prenatal appointment and the date of the first appointment. Prenatal care utilization was measured by using the Kessner index. This index considers the timing of entry in prenatal care, number of prenatal appointments, and gestational age at delivery [30]. 

### 2.3. Statistical Analysis 

We described the study variables using proportions for qualitative variables and means and standard deviations (SD) for quantitative variables. We used the Pearson Chi-square and Kruskal–Wallis tests to assess differences in the distribution of means and percentages. 

We conducted a PCA analysis to ascertain dietary group patterns [24]. According to the similarity of food items, we created 16 food groups (Table 1).

**Figure 2:**
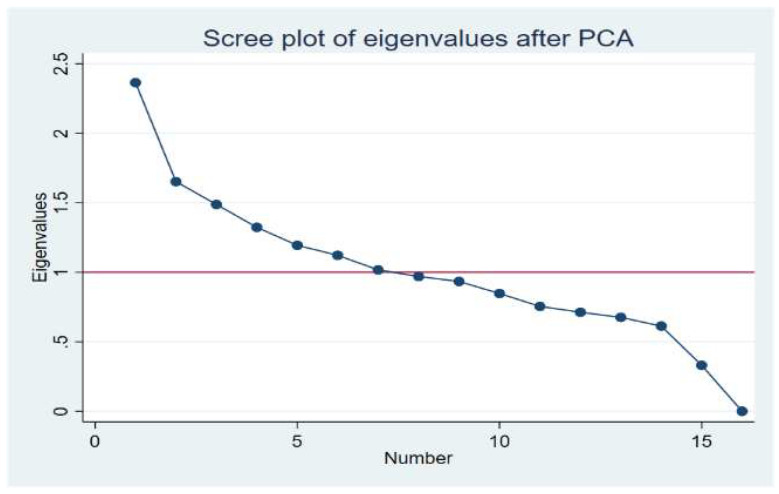
Scree plot of eigenvalues after Principal Components Method (PCA).

We used the Scree plot (Figure 2) and the eigenvalues >1 of the principal components to decide the number of factors to retain. We retained the factors with loadings showing an absolute value ≥0.3. We used these loadings to define the food groups that characterized each dietary pattern.

To explain sampling adequacy and inter-correlation of variables, we used the Kaiser–Meyer–Olkin value and Bartlett’s test of sphericity. Finally, we identified three different dietary patterns explaining 30.6% of the total variance among the food groups included (Table 2).

We used multiple linear regression models to investigate the association between the different dietary patterns (independent variable) with GWG (dependent variable). Crude β-coefficients and adjusted β-coefficients, with their respective 95% confidence intervals (CI), were derived from the fitted univariate and multivariate models. Finally, we used logistic regression models to assess the association between dietary patterns (independent variable) and nutrient adequacy (dependent variable). To control for potential confounding factors in each of the models mentioned previously, multiple logistic and linear models were adjusted for maternal age at pregnancy, social class, Kessner index, and smoking status. Statistical analysis was performed using Stata (15.0, StataCorp L.P. College Station, TX, USA).

## 3. Results

### 3.1. Characteristic of the Study Population

Based on pre-gestational BMI, the overall percentage of women with underweight, normal weight, overweight, and obesity were 12.1%, 57.3%, 22.7% and 8.0%, respectively. Regarding the GWG, 170 (33.8%) women had a reduced GWG. Meanwhile, 128 (25.5%) presented excessive GWG. Table 3 presents maternal sociodemographic, anthropometric, and lifestyle variables stratified by GWG. Women with an excessive GWG had a higher pre-pregnancy BMI (*p* < 0.001), higher mean birth weight, and length of gestation (<0.05) than women with a reduced and adequate GWG.

### 3.2. Dietary Patterns and GWG 

Table 4 presents the crude and adjusted beta (β) coefficients from the univariate and multivariate linear models evaluating the association between each of the two different dietary patterns with an increasing GWG. There was a negative association between GWG and Mediterranean dietary pattern (crude β = −0.06, 95% CI: −0.11, −0.04), whereas a positive association with GWG was found for the Occidental dietary pattern; nevertheless, this association is lost when adjusted for confounders.

### 3.3. Prevalence of Participants with Adequate, Deficient, or Excessive Nutrient Intake According to GWG

Table 5 shows the prevalence of participants with adequate, deficient, or excessive nutrient intake according to their GWG. The vitamins that exhibited the highest deficient intake for all the study participants were vitamins B9 and D. Women with a reduced GWG showed a lower prevalence of vitamin B9 deficient intake than women with an adequate or excessive GWG. In contrast, women with adequate GWG exhibited a higher intake deficit of Vitamin D than women with inadequate or excessive GWG (*p* < 0.05). 

### 3.4. Association between Maternal Dietary Patterns and Nutrient Adequate Intake According to GWG

Table 6 shows the association between nutritional adequacy and dietary patterns according to the GWG status. Independent of GWG, a Mediterranean dietary pattern showed moderate evidence of a higher probability of meeting an adequate intake for dietary fiber; vitamins B9, D, and E; and iodine (*p* < 0.05). 

## 4. Discussion

This is the first study that evaluates the association of maternal dietary patterns and GWG and their association with adequacy nutrient intake in Spanish pregnant women. Independent of maternal GWG, we found that an adequate intake of dietary fiber; vitamins B9, D, and E; calcium; and iodine nutrients was directly related to a classical Mediterranean dietary pattern characterized by a high content of vegetables, olive oil, whole cereals, and nuts. Our findings showed moderate evidence for an association between this healthy dietary pattern and lower GWG trajectories.

Diet quality has been neglected as a risk factor and potential intervention target for inappropriate GWG. Traditionally, the classical nutritional epidemiological approach focused on isolated food groups and/or macronutrient intake instead of the assessment of dietary patterns [31]. However, the role of the overall diet versus individual foods or nutrients provides a more intuitive and objective holistic interpretation of the quality of a woman’s dietary pattern [24]. 

Taking into account that women with healthy dietary intake also have a healthy lifestyle contributing to reducing excess GWG, the position of American Dietetic Association for pregnant women emphasized the adoption of healthy dietary patterns rich in fish and seafood, vegetables, legumes, and vegetable oils, along with engaging in physical activity [32], to prevent inadequate GWG. Accordingly, our study’s main findings support the previous recommendation as we found an association between the classically traditional healthy Mediterranean dietary pattern richer in vegetables, olive oil, and nuts, and lower GWG. In line with our findings, Bassel et al. reported that a Mediterranean-style diet characterized by a high intake of extra virgin olive oil, vegetables, and legumes had a potential role in reducing GWG in British pregnant women [33]. Furthermore, the scientific literature has shown that an increased intake of whole-grain cereal has a positive effect on reducing weight gain not only in a general adult [34] but also in the pregnant women population [35]. Our findings highlight that the Mediterranean dietary pattern, which includes this food group, displays a similar effect. Surprisingly, in our study, dietary patterns dense in energy, such as processed meat and ready-made meals or sweets and dessert patterns, did not show any effect on weight gain. This result contradicts those of other authors that report that a higher energy intake pattern is associated with higher GWG in European pregnant women [36]. Among the reasons that could explain these inconsistent results could be that women who presented a higher GWG (overweight/obese women) reported a healthier dietary intake, overreporting food groups considered as “healthy”, resulting in an information bias, as other authors have extensively communicated [35,36]. Another explanation that could explain the discrepancy in research findings could be that the pregnant women included in our study reported a low intake of these non-healthy products, thus making the identification of this association more difficult.

Pre-pregnancy and pregnancy dietary patterns influence fetal health and the risk of fetal diseases, not only during intrauterine life but also into adulthood. Many nutrients, specifically vitamins and minerals such as vitamin B9 and iron, have been extensively investigated due to their relationship with the development of maternal morbidities and neurodevelopmental disease in babies [37,38]. Even though traditional pregnant counseling has emphasized the consumption of complex prenatal vitamins to provide the necessary amount of some micronutrients [39], Cano-Ibáñez et al. found a positive association between a healthy and diverse dietary pattern with nutrient adequacy in Spanish pregnant women [40]. Accordingly, we found a positive effect of the Mediterranean dietary pattern on nutrient adequacy intake in the present study, independent of GWG. These food groups are considered as “healthy food groups”, typical of dietary patterns such as the Mediterranean diet [41] or Prudent diet [42]. In line with our results, several authors have demonstrated that adherence to both patterns might provide a balanced intake of micronutrients [43,44,45] instead of low energy quantity. The notion is that a food group provides the intake of several nutrients. For example, vitamin B9 is present in leafy vegetables, and dietary fiber can be found in legumes and vegetables.

### Strengths and Limitations

We assessed food intake during pregnancy within 48 h after giving birth; thus, dietary intake during the last trimester may be remembered better than earlier dietary intake during the first trimester. Nevertheless, it has been reported that dietary patterns are stable across pregnancy despite an increased energy intake, with the exception of alcohol intake [46], although recent studies associate very moderate alcohol consumption with lower indices of small for gestational age (SGA) newborns [47]. For this reason, we decided not to include this group in our PCA analysis, as other authors have reported previously [48]. The semiquantitative FFQ has been validated for the Spanish population [18], but self-reporting questionnaires and memory problems could lead to information bias. However, this would more likely cause a non-differential misclassification bias and estimations would tend toward the null. To correct possible errors derived from the FFQ, we excluded participants with energy intakes outside of predefined limits [17], and we used the residual method to adjust for food intake for energy intake. However, although the FFQ specifies the usual portion size as part of the question on frequency, it might not be the ideal tool to measure micronutrient intake and is not validated for this specific population group, although it has been used previously in pregnant women [14]. For this reason, we considered that intake was adequate only when the intake reached at least 2/3 of the recommendations proposed by EFSA for pregnant women, correcting the possible bias introduced by the FFQ and assuming, in any case, that the inadequate micronutrient intake (deficit intake or excessive intake) would be higher than the estimated figures [27]. 

Furthermore, we cannot exclude a potential reverse causation bias in the association between the independent variable with the assessed outcomes due to the study design. Finally, residual confounding that might affect GWG and nutrient intake, such as physical activity or other unmeasured socioeconomic factors, cannot be discarded, even if we have gathered data on the relevant confounders in nutritional epidemiology and adjusted for them in the multivariate analyses.

Notwithstanding the above limitations, our study includes several strengths reinforcing the validity and consistency of the findings obtained. The inclusion of a large representative sample (533 healthy pregnant women), from a reference population of around 120,000 healthy pregnant women providing exhaustive and specific information on dietary intake, is a strength of this study. The use of dietary patterns instead of single food or nutrients is another strength. Dietary patterns are more exhaustive as specific potential factors related to GWG than just single isolated foods. Dietary patterns are more intuitive and objective to determine women’s overall dietary intake during pregnancy [24].

Furthermore, our PCA results explained the 30.6% of the total variance among food groups, and the analysis to derive dietary patterns was based on well-established criteria. We also included a considerable amount of information collected using a standardized protocol that reduces information bias regarding reported food intakes, sociodemographic characteristics, and lifestyles. Finally, to the extent of our knowledge, this report is the first study in Spain assessing the association between GWG with maternal dietary patterns and adequate micronutrient intake.

## 5. Conclusions

In summary, we found that a healthy dietary food intake pattern, compatible with a Mediterranean diet, is associated with an adequate GWG during pregnancy and better nutrient adequacy. Pregnancy can be considered a window of opportunity for promoting healthy habits, as women are more willing to adopt healthier dietary habits during this time. Therefore, our findings support the promotion of healthy dietary habits based on a Mediterranean diet (characterized by a high intake of vegetables, nuts, whole cereals, and olive oil) during pregnancy. Furthermore, counseling and promoting the Mediterranean diet during antenatal visits could offer a sustainable and practical strategy in order to control GWG and ensure adequate nutrient intake during this critical fetal developmental period. Moreover, it could also be an important public health measure with implications that might span over a woman’s life course.

## Figures and Tables

**Table 1:** Food groupings used in factor analysis.

Food Groups	Food Subgroups
**Vegetables**	(1) Green leafy vegetables: spinach, cruciferous, lettuce, green beans, eggplant, peppers, and asparagus; (2) Orange and yellow vegetables: tomatoes, carrots, and pumpkin; (3) Mushrooms.
**Fruits**	Dried fruit, canned fruit, and fresh fruit
**Dairy Products**	(1) Milk: low fat and high fat; (2) Yogurt: low fat and high fat; (3) Cheese: low fat and high fat.
**Whole Cereals**	Whole grain: bread, pasta, rice, and whole breakfast cereals
**Refined Cereals**	Refined grain: bread, pasta, and rice
**Meat**	(1) Red meats: beef, lamb, and organ meats; (2) White meats: poultry and rabbit.
**Meat products**	Hamburger, sausages, and other processed meats
**Fish**	White fish, oily fish, canned fish, and shellfish/seafood
**Sweets and desserts**	Biscuits, cakes, and cookies
**Olive oil**	Olive oil
**Hydrogenated oil**	Butter, margarine, and solid oil
**Potatoes**	Cooked and fried potato
**Legumes**	Peas, beans, lentils, and chickpeas
**Nuts**	Almonds, nuts, pistachios, and other nuts
**Eggs**	Eggs
**Ready-mademeals**	Pizza, soup, lasagna, meatballs, sauces, and other ready-made meals

**Table 2:** Factor loadings for two main dietary patterns derived from a principal component analysis.

Foods/Food Groups	Occidental Dietary Pattern	Mediterranean Dietary Pattern
**Meat**	−0.147	0.237
**Meat products**	0.416	0.376
**Fish**	−0.352	0.263
**Dairy Products**	−0.075	0.063
**Vegetables**	−0.309	0.740
**Whole Cereals**	−0.183	0.504
**Refined Cereals**	0.054	−0.618
**Fruits**	−0.373	0.043
**Nuts**	−0.059	0.323
**Legumes**	−0.050	0.224
**Potatoes**	0.342	0.319
**Olive oil**	−0.005	0.315
**Sweets and desserts**	0.401	−0.128
**Hydrogenated oil**	0.314	−0.092
**Eggs**	0.096	0.032
**Ready-made meals**	0.373	0.207

The cumulative variance contribution rate is 30.6%. Values > 0.3 are factor loading of significant relevance.

**Table 3:** Description of the study population characteristics in the study (*n* = 503).

	Reduced GWG		Adequate GWG		Excessive GWG		*p*-Value
	*n* = 170		*n* = 205		*n* = 128		
**Age in years, mean (SD)**	31.6	(5.5)	31.9	(5.3)	31.0	(4.8)	0.076
**Pre-pregnancy BMI, mean (SD)**	23.3	(3.9)	23.6	(4.0)	25.5	(4.2)	<0.001
**Pre-pregnancy BMI, *n* (%)**							<0.001
Underweight (<18.5 Kg/m^2^)	19	(11.2)	31	(15.1)	11	(8.6)	
Normal weight (18.5–24.9 Kg/m^2^)	121	(71.2)	120	(58.5)	47	(36.7)	
Overweight (25–29.9 Kg/m^2^)	22	(12.9)	40	(19.5)	52	(40.6)	
Obesity (≥30 Kg/m^2^)	8	(4.7)	14	(6.8)	18	(14.1)	
**GWG (kg), mean (SD)**	8.2	(2.9)	12.5	(2.5)	17.3	(3.6)	<0.001
**Birth weight (g), mean (SD)**	3310.5	(379.1)	3436.5	(384.8)	3465.2	(341.7)	<0.001
**Length of gestation (weeks), mean (SD)**	39.4	(1.2)	39.5	(1.2)	39.8	(1.2)	0.013
**Marital status, *n* (%)**							0.312
Singled, never married	15	(8.8)	11	(5.4)	14	(10.9)	
Married	115	(67.7)	147	(71.7)	80	(62.5)	
Couple	40	(23.5)	47	(22.9)	34	(26.6)	
**Educational level, *n* (%)**							0.173
Primary	31	(18.2)	33	(16.1)	26	(20.3)	
Secondary (unfinished)	10	(5.9)	12	(5.9)	4	(3.1)	
Secondary (completed)	50	(29.4)	81	(39.5)	54	(42.2)	
University	79	(46.5)	79	(38.5)	44	(34.4)	
**Smoking during pregnancy, *n* (%)**	14	(8.2)	35	(17.1)	29	(22.7)	0.002
**Kessner index (prenatal care), *n* (%)**							0.396
Adequate	80	(47.1)	95	(46.3)	71	(55.5)	
Intermediate	66	(38.9)	74	(36.1)	38	(29.7)	
Inadequate	24	(14.1)	36	(17.6)	19	(14.8)	

Abbreviations: (SD): standard deviation; BMI: body mass index. Pearson chi-square test and Kruskal–Wallis test were performed for evaluating differences in categorical and continuous variables, respectively.

**Table 4:** Multivariable regression models for the association between dietary patterns and gestational weight gain (GWG) (*n* = 503).

Dietary Pattern	GWG			
	Crude β-Coefficients	(95% CI)	Adjusted β-Coefficients ^a^	(95% CI)
**Occidental dietary pattern**	0.02	(−0.05, 0.04)	0.08	(−0.04, 0.05)
**Mediterranean dietary pattern**	−0.06	(−0.11, −0.04)	−0.05	(−0.01, 0.01)

Crude β-coefficients: crude β-coefficients, adjusted β-coefficients ^a^: adjusted β-coefficients. ^a^ Adjusted for age of parity, social class, Kessner index, and smoking habits.

**Table 5:** Prevalence of participants with an adequate, deficient, or excessive intake of nutrients according to 2/3 EFSA DRIs stratified by GWG.

	Reduced GWG			Adequate GWG			Excessive GWG			
Nutrient	*n* = 170			*n* = 205			*n* = 128			
	DI ^a^	AI ^b^	EI ^c^	DI ^a^	AI ^b^	EI ^c^	DI ^a^	AI ^b^	EI ^c^	*p*-Value
**Dietary fiber (g/day)**	6.0	94.1	-	9.3	90.7	-	9.4	90.6	-	0.413
**Vitamin A (µg/day)**	0	58.2	41.8	0.5	59.5	40.0	0.8	62.5	36.7	0.756
**Vitamin B9 (µg/day)**	45.3	50.0	4.7	58.0	41.0	1.0	54.7	40.6	4.7	0.034
**Vitamin B12 (µg/day)**	0	100.0	-	0.5	99.5	-	1.6	98.4	-	0.215
**Vitamin D (µg/day)**	82.4	17.7	0	89.8	10.2	0	80.5	19.5	0	0.037
**Vitamin E (mg/day)**	5.9	94.1	0	2.9	97.1	0	3.1	96.9	0	0.293
**Calcium (mg/day)**	0.6	87.1	12.4	0.5	88.8	10.7	0.8	82.8	16.4	0.651
**Magnesium (mg/day)**	0	0	100.0	0	0	100.0	0	0	100.0	-
**Iodine (µg/day)**	9.4	60.6	30.0	7.8	53.7	38.5	10.9	50.0	39.1	0.299
**Potassium (mg/day)**	0.6	99.4	-	0	100.0	-	1.6	98.4	-	0.197
**Selenium (µg/day)**	1.2	98.8	0	1.5	98.5	0	3.1	96.1	0.8	0.314

Intake (I): ^a^ deficient intake (DI), ^b^ adequate intake (AI), and ^c^ excessive intake (EI). Values are % unless otherwise indicated. Pearson chi-square test was used in order to ascertain differences between groups. There is no Upper-Level intake (UL) in the micronutrient assessed.

**Table 6:** Multivariate logistic regression of association between nutrient adequacy and dietary patterns according to GWG.

	Reduced GWG OR (95% CI)	Adequate GWG OR (95% CI)	Excessive GWG OR (95% CI)
Dietary fiber			
**Occidental dietary pattern**	0.8 (0.42, 1.35)	0.6 (0.39, 1.03)	0.4 (0.15, 1.86)
**Mediterranean dietary pattern**	3.1 (1.37, 7.07)	1.6 (0.96, 2.59)	1.4 (0.72, 2.57)
Vitamin A			
**Occidental dietary pattern**	0.9 (0.67, 1.10)	0.7 (0.54, 0.87)	0.6 (0.44, 0.82)
**Mediterranean dietary pattern**	1.1 (0.80, 1.36)	1.2 (0.94, 1.51)	1.1 (0.78, 1.43)
Vitamin B9			
**Occidental dietary pattern**	0.5 (0.44, 0.81)	0.6 (0.44, 0.77)	0.8 (0.56, 1.08)
**Mediterranean dietary pattern**	1.6 (1.22, 2.21)	2.1 (1.56, 2.79)	1.7 (1.26, 2.36)
Vitamin D			
**Occidental dietary pattern**	0.5 (0.32, 0.87)	0.74 (0.46, 1.19)	0.91 (0.61, 1.38)
**Mediterranean dietary pattern**	4.4 (2.50, 7.68)	4.89 (2.72, 8.77)	3.02 (1.84, 4.96)
Vitamin E			
**Occidental dietary pattern**	1.06 (0.62, 1.82)	1.1 (0.54, 2.06)	1.00 (0.40, 2.51)
**Mediterranean dietary pattern**	2.7 (1.26, 5.72)	1.26 (0.64, 2.50)	2.75 (0.90, 8.34)
Calcium			
**Occidental dietary pattern**	0.8 (0.56, 1.09)	0.8 (0.59, 1.15)	0.82 (0.65, 1.04)
**Mediterranean dietary pattern**	1.4 (0.92, 2.13)	1.20 (0.85, 1.70)	1.15 (0.80, 1.67)
Iodine			
**Occidental dietary pattern**	0.8 (0.65, 1.12)	1.2 (0.93, 1.48)	1.0 (0.78, 1.35)
**Mediterranean dietary pattern**	1.3 (0.97, 1.68)	1.3 (1.02, 1.63)	1.1 (0.91, 1.43)

The multivariable model was adjusted for age of parity, social class, Kessner index, and smoking habits.
